# Effect of COVID-19 on the mental health care of older people in Canada

**DOI:** 10.1017/S1041610220000708

**Published:** 2020-04-24

**Authors:** Alastair J. Flint, Kathleen S. Bingham, Andrea Iaboni

**Affiliations:** Centre for Mental Health, University Health Network and the Department of Psychiatry, University of Toronto, Toronto, Canada

Like other developed countries, Canada has an aging population, although less than most other G7 nations. Of a total population of 35 million people, 16.9% are aged 65 years or older (Statistics Canada, 2017a). Although the virus responsible for coronavirus disease 2019 (COVID-19) can affect persons of any age, older adults are particularly vulnerable to serious infection and death (Verity *et al.*, 2020) because of an age-related decline in immune function and the likelihood of having more preexisting health conditions than younger individuals (Nikolich-Zugich *et al.*, 2020). Moreover, although only 5% of Canadian seniors live in long-term care homes (LTCH) (nursing homes) (Statistics Canada, 2017b), residents of LTCH have accounted for a disproportionate number of infections and deaths due to COVID-19 in Canada (Hsu and Lane, 2020). As of mid-April 2020, more than 40% of COVID-19-related deaths in Canada had occurred in LTCH (Hsu and Lane, 2020).

Canada has a publicly funded, universally accessible, national health care system (“Medicare”), which provides Canadian citizens and permanent residents with prepaid medical care and hospital care. In addition, most drug costs, the basic costs of long-term care, and limited home care services are government funded for persons aged 65 years or older. As a result, there are no direct financial barriers to older Canadians’ access to health care during the COVID-19 pandemic. In addition, as of April 12, Canada has been spared the massive disruption to its health care system that has occurred in some other jurisdictions such as Italy and parts of the U.S.A. (Weeks, 2020) and so mental health services, which may be vulnerable to such disruptions, have remained relatively intact.

## Problems faced during the past several months

In 2003, Canada (primarily Toronto) was affected by an outbreak of severe acute respiratory syndrome coronavirus 1 (SARS). Lessons learned from SARS resulted in positive changes at provincial and federal levels of government in preparing for future outbreaks of disease and pandemics, not least of which was a better funded, more integrated, and more responsive public health system (Tam, 2018). As a result, following the outbreak of COVID-19, Canada was more prepared than some other countries to prevent the spread of the virus. Nevertheless, the ease of transmission of the COVID-19 virus and its potential to kill healthy people (Verity *et al.*, 2020) have led to significant changes within Canadian society that have affected the delivery of mental health care and have the potential to affect the mental health of Canadians, including its senior citizens.

The most significant societal change to date has been so-called “social distancing,” which has led to government-mandated closure of nonessential businesses, schools, sports and recreational facilities, and places of worship; prevents public gatherings; and advises individuals to stay in their home except for essential activities and brief periods of exercise in their neighborhood. This policy has resulted in a number of changes to the delivery of mental health care. Most outpatient care is now delivered virtually, either by video or telephone. Based on anecdotal experience, there has been a fairly seamless transition in the provision of care for persons who live at home, particularly since Medicare fees were adjusted to compensate physicians for provision of telephone care, which is not typically reimbursed.

Canada has been successful thus far in limiting the burden of COVID-19 on its hospital system. However, this positive news belies a crisis in the LTC system, where hundreds of facilities across Canada have experienced outbreaks of infection (Hsu and Lane, 2020). This has made it challenging to provide mental health care to residents of LTCH, where psychogeriatric staff would previously visit the homes to provide care. Among the challenges are the limited availability of portable telemedicine technology in some LTCH and the difficulty in providing virtual care to residents who are in isolation because of infection. Moreover, social distancing practices in LTCH (including a prohibition on all but essential visitors) now place even more demands on the time of busy frontline staff, who may therefore be less available to participate in the process of providing psychogeriatric assessment and care. Finally, given the disproportionate incidence of COVID-19 infections in LTCH, frontline staff are at higher risk of being infected with the virus, resulting in increased psychological distress among staff, as well as staff shortages due to sickness and absenteeism. Staff shortages may be further exacerbated by the recent decision by some provincial governments to stop LTC staff from working at multiple facilities in an effort to prevent the spread of the virus (Van Evra, 2020).

The need for social distancing has also affected the provision of outpatient electroconvulsive therapy (ECT). Psychiatrists have argued that ECT is an essential service, both as an acute treatment for persons with serious mental illness, including those at high risk of suicide, as well as preventing relapse and recurrence of serious mental illness that cannot be satisfactorily managed with other types of treatment. Nevertheless, provision of ECT in some hospitals has been suspended or significantly curtailed, in order to minimize the risk of transmission of the coronavirus from the community to hospitals.

With respect to other services, the policy of social distancing has led to the suspension of community groups and day programs for seniors. Homecare services continue, but at reduced capacity and with care providers wearing masks and gloves. However, shortages of personal protective equipment in Canada, especially among health care workers in LTCH and the community, may result in some homecare workers reusing masks and gloves from one home to the next, increasing the risk of spread of infection. Some seniors have elected to suspend their home care services, to avoid the risk of being infected.

Research data are not yet available on the effect of COVID-19, and the measures used to prevent its spread, on the mental health of older Canadians. As noted, the publically funded and universally accessible health care system and telemedicine services have allowed continuity of most outpatient care for those individuals already receiving mental health care, notwithstanding the disruption to services provided to LTCH. However, it is probable that social distancing will lead to less frequent contact by older adults with their family physicians, who are the frontline for treating mental health problems in Canada and the gatekeepers for most outpatient psychiatric referrals. As a result, more incident cases of mental disorder may go undetected and untreated. Social distancing may also lead to an increased sense of isolation and loneliness, which are risk factors for the development of depression and cognitive impairment (Choi *et al*., 2015; Read, 2020).

## Solutions being implemented and those being considered

The most prevalent solution has been to expand the use of virtual care, either by video or by telephone for individuals who do not have video capability or prefer to not use video. Canada has a well-developed telemedicine infrastructure, so the wholesale transition to virtual care of outpatients, to protect the health of providers and patients, happened almost overnight. Access to pharmacies has been unhindered, with many pharmacies expanding their home delivery service. Pharmacies have been restricting quantities of drugs to one month at a time, in an attempt to forestall drug shortages, should there be production issues in the future. Mental health and caregiver organizations have expanded their online presence, with information about the potential effect of the pandemic and related public health measures on mental health, as well as strategies for coping, including links to web-based counseling and advice on how to make the best use of technology. Peer-support groups, such as those affiliated with Alzheimer’s organizations and addiction services, have moved activities online.

Some cities have rented additional space for shelters for the homeless, a group that includes older persons with chronic mental illness and substance use disorders, to facilitate social distancing and help minimize the spread of the coronavirus among this vulnerable group (The Canadian Press, 2020).

With respect to hospital-based care, infection protocols are well established, partly the result of lessons learned from the 2003 SARS outbreak. Social distancing measures and use of personal protective equipment by staff have been implemented on inpatient units. Recreational therapists have found creative ways to engage inpatients, in the context of social distancing. In cases where ECT is given, strict infection protocols have been introduced, given the potential for some of the processes associated with ECT (high-flow oxygen, bag-valve mask ventilation, potential for coughing by the patient during recovery) to lead to aerosol spread of the virus.

Most universities and hospitals have suspended on-site research that is deemed to be nonessential. Many institutional review boards have allowed for clinical research activities to be continued remotely, so as to minimize risk of infection of research participants and research staff, but allow for the ongoing collection of data to maintain the integrity and safety of studies. Research groups are currently amending protocols and instruments, to ensure that remotely collected data are reliable and valid; this is a particular challenge in studies that perform detailed neuropsychological testing.

The long-term care sector is in need of innovative solutions to provide psychiatric care to residents and support for staff. LTCH are typically understaffed (Hsu and Lane, 2020), and many staff do not have the knowledge and skills to adequately manage psychiatric symptoms and problematic behaviors. In addition, some facilities have limited access to smoking areas, which creates a challenge in managing residents with chronic mental health conditions and nicotine dependence. LTCH rely on the resources and expertise of psychogeriatric outreach teams to provide guidance in managing residents with dementia, as well as older individuals with serious and persistent mental illness. In lieu of providing on-site support during the COVID-19 outbreak, psychogeriatric teams are exploring ways to provide impactful digital support. For example, digital access to behavioral support specialists who are on call and can provide rapid consultation is one potential model. Tablets could be used by LTCH staff, as well as by family members, to observe, support, and interact with residents who are in physical isolation because of infection, although the current challenges are availability of devices and uneven wi-fi connection.

## Outlook and suggestions for the future

The immediate outlook depends on how quickly and effectively transmission of the virus can be contained and whether widespread disruption of the health care system in Canada can be avoided. As of mid-April, Canada has sufficient hospital bed capacity and intensive care unit capacity to manage. However, should the acute care sector be overwhelmed by cases of COVID-19 infection, it is possible that there will be downstream effects that adversely affect the provision of psychogeriatric care.

The current model of widespread provision of virtual care will remain in place until social distancing rules are significantly relaxed, which will likely depend on the development of a vaccine, and possibly efficacious therapeutics, to manage the COVID-19 pandemic (Powell, 2020). However, the current crisis will hopefully lead to more sustained integration of virtual care into geriatric psychiatry practice in the future, leading to more person-centered care and improved access to care.

The COVID-19 pandemic has shone a light on the need to reform the long-term care sector in Canada. Most LTCH are privately owned and operated but regulated by provincial governments. LTCH are underresourced and overregulated (Dijkema and Wolfert, 2019). Many personal support worker positions are part-time contracts, resulting in workers taking positions at multiple facilities (Pedersen and Mancini, 2020). By 2035, double the number of long-term care beds will be needed – an additional 200,000 beds (Gibbard, 2017). The current crisis has highlighted the need for more and better-trained staff, better job security for personal support workers, more innovative ways of delivering care, and greater government oversight that is provided in a less bureaucratic way.

Finally, as in other countries, the economic toll of the pandemic in Canada is widespread and profound. Health spending in Canada represents close to 40% of total provincial and territorial program expenditures (Canadian Institute for Health Information, 2019). It remains to be seen whether, and to what extent, the longer term funding of heath care, including seniors’ mental health care, will be diminished by this economic fallout.
